# Saringosterol from *Sargassum fusiforme* Modulates Cholesterol Metabolism and Alleviates Atherosclerosis in ApoE-Deficient Mice

**DOI:** 10.3390/md19090485

**Published:** 2021-08-26

**Authors:** Ying Yan, Zhoumin Niu, Boyang Wang, Shangge Zhao, Chao Sun, Yuting Wu, Yuying Li, Hao Ying, Hongbing Liu

**Affiliations:** 1 CAS Key Laboratory of Nutrition, Metabolism and Food Safety, Shanghai Institute of Nutrition and Health, University of Chinese Academy of Sciences, Chinese Academy of Sciences, Shanghai 200031, China; yanying2017@sibs.ac.cn (Y.Y.); niuzhoumin2020@sibs.ac.cn (Z.N.); sunchao@sibs.ac.cn (C.S.); ytwu@sibs.ac.cn (Y.W.); liyuying@sibs.ac.cn (Y.L.); 2 Key Laboratory of Marine Drugs, Chinese Ministry of Education, School of Medicine and Pharmacy, Ocean University of China, Qingdao 266003, China; wangboyang@stu.ouc.edu.cn (B.W.); zhaoshangge@stu.ouc.edu.cn (S.Z.); 3 Laboratory for Marine Drugs and Bioproducts, Pilot National Laboratory for Marine Science and Technology (Qingdao), Qingdao 266237, China; 4 Key Laboratory of Food Safety Risk Assessment, Ministry of Health, Beijing 100021, China

**Keywords:** saringosterol, liver X receptor, cholesterol metabolism, atherosclerosis, *Sargassum fusiforme*

## Abstract

Dysregulation of cholesterol homeostasis is a major risk factor of atherosclerosis, which can lead to serious health problems, including heart attack and stroke. Liver X receptor (LXR) α and β are transcription factors belonging to the nuclear receptor superfamily, which play important roles in cholesterol homeostasis. Selectively activating LXRβ provides a promising strategy for the treatment of atherosclerosis. Here, we employed atherosclerotic apoE-knockout mice to evaluate the effects of saringosterol, a phytosterol with potent and selective action for LXRβ, which we identified previously in edible marine seaweed *Sargassum fusiforme*. We found that saringosterol treatment reduced the atherosclerotic plaque burden without having undesirable adverse hepatic effects in apoE-deficient mice fed an atherogenic diet. Meanwhile, reduced serum levels of cholesterol, accompanied by altered expression of LXR-regulated genes involved in cholesterol absorption, transport, efflux, excretion, and elimination, were observed in apoE-knockout mice after saringosterol treatment. Together, our study not only establishes saringosterol as an effective cholesterol-lowering and anti-atherogenic phytosterol but also provides insights into the underlying mechanism.

## 1. Introduction

Dysregulation of cholesterol homeostasis is a major risk factor of atherosclerosis, which is a leading cause of morbidity and mortality worldwide. Atherosclerotic plaques, arising from an imbalance in lipid metabolism, are characterized by the accumulation of abnormal amounts of cholesterol in the artery wall [1]. The accumulation of cholesterol in macrophage-derived foam cells is a critical step in the development of atherosclerosis, while elevated plasma cholesterol levels can promote atherosclerosis [2,3]. It has been well-accepted that proper control of cellular and systemic cholesterol homeostasis is crucial in preventing atherogenesis.

The liver X receptors (LXRs) (LXRα and LXRβ) are ligand-activated transcription factors belonging to the nuclear receptor superfamily [4,5]. As LXRs regulate the expression of many genes involved in cholesterol absorption, transport, efflux, excretion, and elimination, LXRs are essential for maintaining cholesterol homeostasis. LXR activation can prevent the development and progression of atherosclerosis through lowering the blood cholesterol levels by multiple mechanisms [6,7]. In macrophages, LXR promotes cholesterol efflux, the initial step of reverse cholesterol transport (RCT), via inducing the expression of ATP-binding cassette subfamily A member 1 (ABCA1) and subfamily G member 1 (ABCG1) [8,9]. LXR also reduces the cholesterol uptake by inducing the transcription of inducible degrader of the LDL receptor (IDOL), which contributes to the degradation of the LDL receptor (LDLR) [10]. In the liver, LXR promotes the cholesterol catabolism and excretion via increasing the expression of cytochrome P450 family 7 subfamily A member 1 (CYP7A1) and ATP-binding cassette subfamily G members 5 (ABCG5) and 8 (ABCG8) [11,12]. In the intestine, LXR reduces the dietary cholesterol absorption from the intestinal lumen, increases cholesterol efflux from enterocytes to circulation and HDL formation, and promotes fecal cholesterol excretion by decreasing the expression of Niemann–Pick C1-like protein 1 (NPC1L1) and increasing the expression of ABCA1, ABCG5, and ABCG8, respectively [13,14].

Given the beneficial effects of LXR activation in animal models of atherosclerosis, LXR has been considered as a potential drug target. It has been reported that synthetic LXR agonists such as T0901317 and GW3965 are able to attenuate the progression of atherosclerosis [15,16]. However, these two pan-LXR agonists have undesirable effects, as they can lead to hepatic steatosis and hypertriglyceridaemia [17]. Growing evidence suggests that the lipogenic activity of LXRs is mainly attributed to the activation of sterol regulatory element-binding protein-1c (SREBP-1c) mediated by LXRα in the liver [18,19]. Thus, it has been proposed that LXRβ-selective agonists may have fewer adverse effects, as they can exert anti-atherosclerosis effects without promoting hepatic lipid accumulation [20].

*Sargassum fusiforme* is a kind of brown edible algae that is widely distributed on the seashores of China, Korea, and Japan as a therapeutic for thousands of years [21]. Saringosterol, a phytosterol extracted from *Sargassum fusiforme*, has been shown to exhibit anti-tubercular activity by inhibiting the growth of *Mycobacterium tuberculosis* [22], as well as antidepressant-like effects [23], anti-obesity activity [24], and the effect of preventing cognitive decline [25]. Furthermore, we previously demonstrated that saringosterol has a potent and selective action for LXRβ in vitro, and is a potentially natural cholesterol-lowering agent [26]. However, there is no report to date on the anti-atherosclerotic effect of saringosterol in the mouse model of atherosclerosis. ApoE-knockout (ApoE^−/−^) mice are characterized by high remnant lipoprotein and low HDL levels and consequently develop spontaneous hyperlipidemia and atherosclerosis, making themselves widely used to evaluate the impact of therapeutic agents on atherogenesis. In this study, the effects of saringosterol in ApoE^−/^^−^ mice were investigated. We found that saringosterol could affect the expression of LXR-regulated genes involved in cholesterol homeostasis; meanwhile, it reduced the serum cholesterol levels and attenuated the atherosclerotic progression without showing adverse effects.

## 2. Results

### 2.1. Saringosterol Treatment Reduces Atherosclerotic Plaques in ApoE^−/−^ Mice

**Figure 1:**
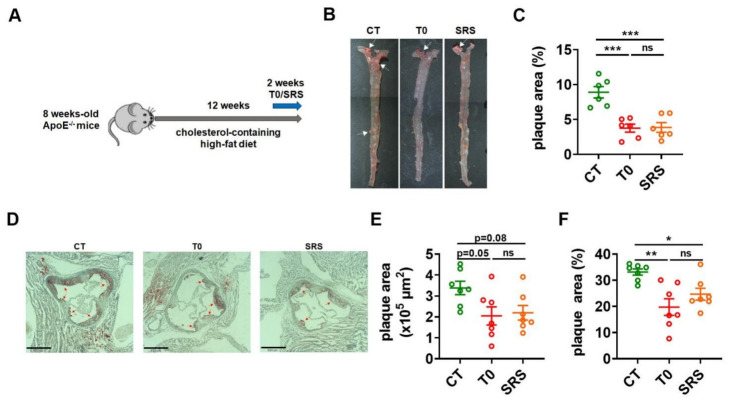
Effects of saringosterol on the formation of atherosclerotic plaques in ApoE^−/−^ mice. (**A**) Schematic diagram of experimental setup. ApoE^−/−^ mice were all fed a high-fat diet with added cholesterol for 12 weeks. Mice were orally administered saringosterol or T0901317 once daily for the last 2 weeks as indicated. (**B**,**C**) Representative images of en face Sudan IV staining of aortas (**B**) and quantification of the plaque areas of aortas (**C**). Data were presented as percentages of the plaque area to total aortic area (*n* = 7). (**D**–**F**) Representative image of Oil Red O staining of an aortic lesion (Bars: 520 μm. Magnification: 4×) (**D**) and quantification of the lesion areas (**E**,**F**). Data were presented as plaque areas (**E**) and percentages of the plaque area (**F**) (*n* = 7). CT, control; T0, T0901317; and SRS, Saringosterol. Means ± SEM are shown. The statistical differences in mean values were assessed via one-way ANOVA analysis followed by Tukey’s multiple comparison test. * *p* < 0.05, ** *p* < 0.01 and *** *p* < 0.001. ns, not significant.

To study the effects of saringosterol on the formation of atherosclerotic plaques, ApoE^−/−^ mice were randomly divided into three groups and fed with a high-fat diet with added cholesterol, followed by 2 weeks of saringosterol (SRS), T0901317 (T0), or vehicle (CT) treatment (Figure 1A). En face analysis of aortas via Sudan IV staining revealed a marked reduction of atherosclerotic lesion area in ApoE^−/−^ mice receiving saringosterol treatment (SRS group) compared to control mice treated with vehicle (CT group) (Figure 1B,C). As expected, reduced en face aortic lesions were also observed in ApoE^−/−^ mice receiving T0901317 treatment (T0 group) (Figure 1B,C). The atherosclerotic plaque burden was further evaluated in the cross-sections of the aortic root via Oil Red O staining. The ApoE^−/−^ mice in SRS group displayed a reduction in plaque area compared to ApoE^−/−^ mice in CT group (Figure 1D–F). These results suggest that saringosterol administration could attenuate the formation of atherosclerotic plaques in atherosclerotic ApoE^−/−^ mice.

### 2.2. Saringosterol Administration Improves Lipid Profiles in ApoE^−/−^ Mice

**Figure 2:**
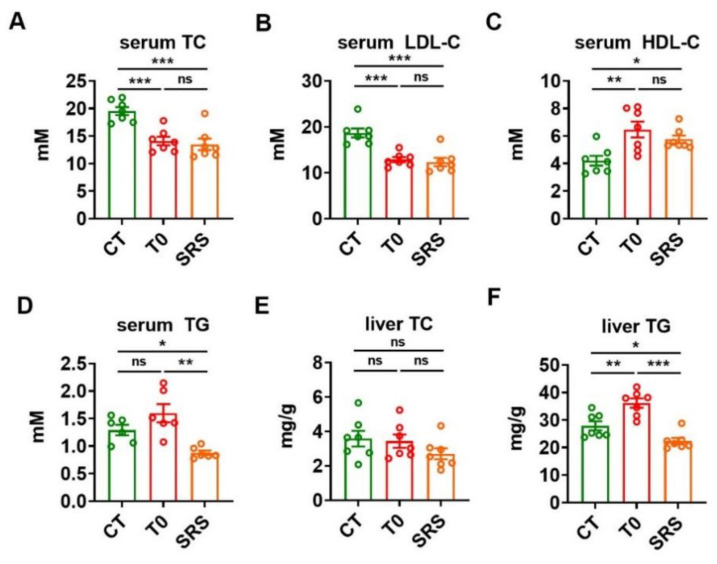
Effects of saringosterol on the serum and hepatic lipid profiles in ApoE^−/−^ mice. (**A**–**D**) Serum levels of total cholesterol (TC) (**A**), LDL-C (**B**), HDL-C (**C**) and triglyceride (TG) (**D**) in ApoE^−/−^ mice after saringosterol or T0901317 treatment (*n* = 7). (**E**,**F**) TC (**E**) and TG (**F**) levels in the liver of ApoE^−/−^ mice after saringosterol or T0901317 treatment (*n* = 7). CT, control; T0, T0901317; and SRS, Saringosterol. Means ± SEM are shown. The statistical differences in mean values were assessed by one-way ANOVA analysis followed by Tukey’s multiple comparison test. * *p* < 0.05, ** *p* < 0.01 and *** *p* < 0.001. ns, not significant.

To examine the effect of saringosterol on lipid homeostasis, we evaluated the levels of cholesterol and triglyceride (TG) in the serum and liver of ApoE^−/−^ mice. The ApoE^−/−^ mice in either SRS group or T0 group showed a significant decrease in serum total cholesterol (TC) compared to ApoE^−/−^ mice in CT group (Figure 2A). Moreover, serum low-density lipoprotein cholesterol (LDL-C) levels were reduced in ApoE^−/−^ mice in both SRS and T0 groups compared to that in the CT group (Figure 2B). Additionally, serum high-density lipoprotein cholesterol (HDL-C) levels were increased in ApoE^−/−^ mice in both SRS and T0 groups compared to CT group (Figure 2C). As expected, T0901317 administration tended to increase the serum TG levels in these ApoE^−/−^ mice. In contrast, saringosterol treatment resulted in a significant decrease in serum TG levels in atherosclerotic ApoE^−/−^ mice (Figure 2D). No significant differences in liver cholesterol levels were observed among these three groups (Figure 2E). As expected, T0901317 treatment increased the TG levels in the liver of atherosclerotic ApoE^−/−^ mice. In contrast, saringosterol treatment reduced rather than increased the TG content in the liver of these ApoE^−/−^ mice (Figure 2F). These results indicate that saringosterol treatment not only could improve serum cholesterol profile but also had favorable rather than adverse effects on serum and hepatic TG levels in ApoE^−/−^ mice.

### 2.3. Saringosterol Treatment Modulates Cholesterol Metabolism in Macrophagess

**Figure 3:**
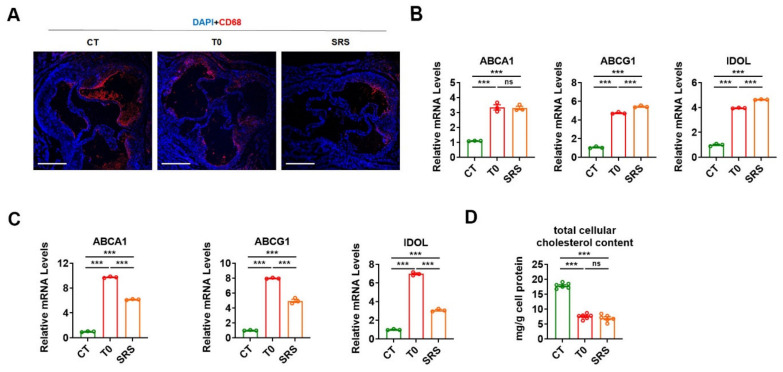
Saringosterol effects on the cholesterol metabolism in macrophages of ApoE^−/−^ mice. (**A**) Macrophage content was determined by staining with anti-macrophage marker anti-body (CD68) (red) in aortic root of ApoE^−/−^ mice treated with saringosterol or T0901317. Bars: 520 μm. Magnification: 4×. (**B**) Relative mRNA levels of ABCA1, ABCG1, and IDOL in peritoneal macrophages harvested from ApoE^−/−^ mice treated with saringosterol or T0901317 (*n* = 3). (**C**) Relative mRNA levels of ABCA1, ABCG1, and IDOL in RAW264.7 macrophage-derived foam cells treated with saringosterol or T0901317 (*n* = 3). (**D**) Total cellular cholesterol content was analyzed in RAW264.7 macrophage-derived foam cells treated with saringosterol or T0901317 (*n* = 3). CT, control; T0, T0901317; and SRS, saringosterol. Means ± SEM are shown. The statistical differences in mean values were assessed by one-way ANOVA analysis followed by Tukey’s multiple comparison test. *** *p* < 0.001. ns, not significant.

As the accumulation of lipid-laden macrophage foam cells in the intima of inflamed arteries is a hallmark of atherosclerosis, the prevalence of macrophages within the atherosclerotic plaque was evaluated in ApoE^−/−^ mice after treatment. As expected, either saringosterol or T0901317 treatment led to a decrease in CD68 staining within the atherosclerotic plaques in the aortic root (Figure 3A). These data, together with the Oil Red O staining results (Figure 1D), suggest that both saringosterol and T0901317 treatment are able to suppress foam cell formation. Accordingly, the mRNA levels of two cholesterol transporters, ABCA1 and ABCG1 (which are LXR target genes that mediate the apoA-I- and HDL-dependent cholesterol efflux in macrophages) were both markedly elevated in the peritoneal macrophages obtained from ApoE^−/−^ mice after either saringosterol or T0901317 treatment (Figure 3B). Additionally, the mRNA expression of IDOL (which is an LXR target gene responsible for LDLR degradation) was increased in the peritoneal macrophages harvested from ApoE^−/−^ mice after either saringosterol or T0901317 treatment (Figure 3B). The regulation of ABCA1, ABCG1, and IDOL mRNA expression by either saringosterol or T0901317 treatment could also be observed in RAW264.7 macrophage-derived foam cells (Figure 3C). These results suggest that saringosterol might be able to promote cellular cholesterol efflux and inhibit cholesterol uptake in macrophages by activating LXR. In line with these results, the total cellular cholesterol content was significantly reduced in RAW264.7 macrophage-derived foam cells after either saringosterol or T0901317 treatment (Figure 3D). Together, our results suggest that saringosterol treatment might enhance RCT and suppress foam cells formation by modulating LXR-mediated cholesterol efflux and uptake in macrophages, thereby decelerating the development and progression of atherosclerosis.

### 2.4. Saringosterol Treatment Affects Cholesterol Metabolism in Liver and Intestine

**Figure 4:**
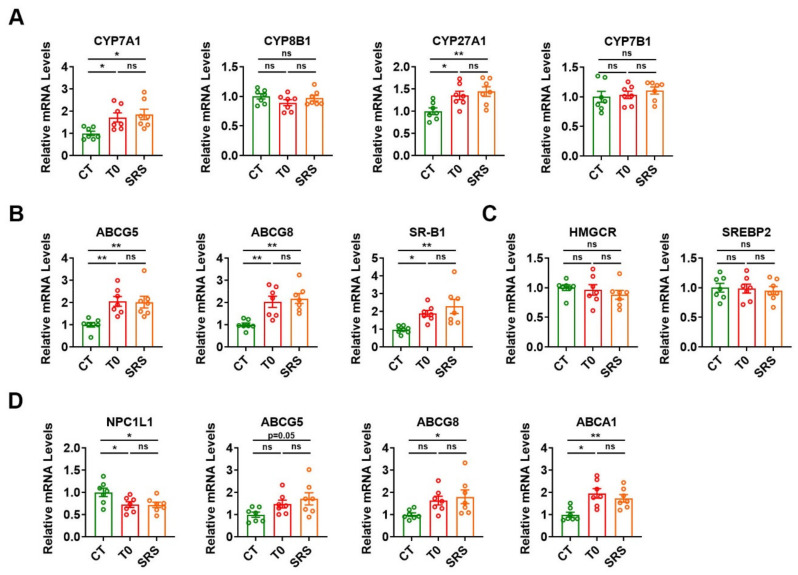
Saringosterol effects on cholesterol metabolism in the liver and intestine of ApoE^−/−^ mice. (**A**–**C**) Relative mRNA levels of genes involved in cholesterol catabolism (CYP7A1, CYP8B1, CYP27A1, and CYP7B1) (**A**), cholesterol efflux (ABCG5 and ABCG8) (**B**), influx (SR-B1) (**B**), synthesis (HMGCR and SREBP2) (**C**) in the liver of ApoE^−/−^ mice treated with Saringosterol or T0901317 (*n* = 7). (**D**) Relative mRNA levels of genes involved in cholesterol absorption (NPC1L1), excretion, and efflux (ABCG5, ABCG8, and ABCA1) in the intestine of ApoE^−/−^ mice treated with Saringosterol or T0901317 (*n* = 7). CT, control; T0, T0901317; and SRS, Saringosterol. Means ± SEM are shown. The statistical differences in mean values were assessed by one-way ANOVA analysis followed by Tukey’s multiple comparison test. * *p* < 0.05, ** *p* < 0.01. ns, not significant.

Since the regulation of cholesterol metabolism by LXR in the liver and intestine also contributes to its cholesterol-lowering and anti-atherogenic effects, we pinpointed the impact of saringosterol on LXR-regulated genes in the liver and intestine of ApoE^−/−^ mice. We first examined the effect of saringosterol on the expression of key enzymes involved in bile acid synthesis, a major pathway for hepatic cholesterol catabolism. The mRNA expression of CYP7A1, a direct LXR target gene encoding the rate-limiting enzyme in bile acid synthesis, was increased in the liver of ApoE^−/−^ mice in SRS group (Figure 4A). Saringosterol treatment upregulated the mRNA levels of CYP27A1, but had no effect on the mRNA expression of CYP8B1 and CYP7B1 (Figure 4A). The mRNA expression of ABCG5 and ABCG8, two LXR target genes that mediate the biliary excretion of cholesterol from the liver, was significantly increased in the liver of ApoE^−/−^ mice in the SRS group (Figure 4B). The mRNA levels of scavenger receptor B type I (SR-B1), which mediates the transfer of HDL-cholesterol to the hepatocytes, were upregulated in the liver of ApoE^−/−^ mice in the SRS group (Figure 4B). In contrast, the mRNA levels of genes involved in cholesterol synthesis, 3-hydroxy-3-methylglutaryl-CoA reductase (HMGCR) and sterol regulatory element-binding protein 2 (SREBP2), were not significantly changed in the liver of ApoE^−/−^ mice after saringosterol treatment (Figure 4C). Similar results were obtained in the liver of ApoE^−/−^ mice after T0901317 treatment. These data indicate that saringosterol might promote the cholesterol elimination from the body through increasing the hepatic cholesterol catabolism and excretion by activating LXR, thereby alleviating the atherosclerosis.

Meanwhile, the mRNA expression of NPC1L1, which is responsible for the dietary cholesterol absorption from the intestinal lumen, was significantly downregulated in the intestine of ApoE^−/−^ mice after saringosterol treatment (Figure 4D). The mRNA levels of ABCG5 and ABCG8, which mediate the cholesterol efflux from enterocytes into the lumen and transintestinal cholesterol excretion, were increased in the intestine of ApoE^−/−^ mice in the SRS group (Figure 4D). The mRNA levels of basolateral ABCA1, which mediates the cholesterol efflux from enterocytes to circulation and HDL formation, were also significantly elevated in the intestine of ApoE^−/−^ mice in the SRS group (Figure 4D). Similar results could be observed in the intestine of ApoE^−/−^ mice after T0901317 treatment (Figure 4D). These findings indicate that saringosterol might also act on intestine and inhibit dietary cholesterol absorption, promote RCT, and enhance trans-intestinal cholesterol excretion by activating LXR, which would also contribute to its cholesterol-lowering and anti-atherosclerotic effects.

### 2.5. Saringosterol Treatment Does Not Worsen Hepatic Steatosis in ApoE^−/−^ Mice

**Figure 5:**
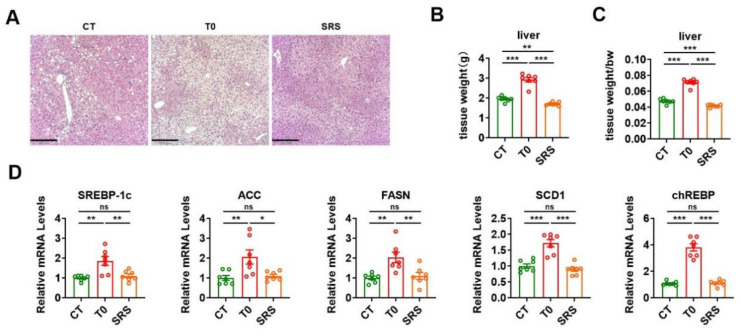
Effects of saringosterol on the lipogenesis in the liver of ApoE^−/−^ mice. (**A**) Representative images of HE staining of the liver of ApoE^−/−^ mice treated with saringosterol or T0901317. Bars: 210 μm. Magnification: 10×. (**B**,**C**) Liver weight (**B**) and ratio of the liver weight to body weight (**C**) in ApoE^−/−^ mice treated with saringosterol or T0901317 (*n* = 7). (**D**) Relative mRNA levels of lipogenic genes in the liver of ApoE^−/−^ mice treated with saringosterol or T0901317 (*n* = 7). CT, control; T0, T0901317; and SRS, saringosterol. Means ± SEM are shown. The statistical differences in mean values were assessed by one-way ANOVA analysis followed by Tukey’s multiple comparison test. * *p* < 0.05, ** *p* < 0.01 and *** *p* < 0.001. ns, not significant.

As saringosterol has a selective action for LXRβ (which may have fewer adverse effects than LXRα agonists), we further evaluated whether saringosterol treatment had a lipogenic effect in the liver of atherosclerotic ApoE^−/−^ mice. Consistent with previous reports and our above results (Figure 2F), H&E staining results suggest that T0901317 treatment could exacerbate the hepatic steatosis in atherosclerotic ApoE^−/−^ mice (Figure 5A). In line with the above findings that the TG levels were reduced in the liver of saringosterol-treated ApoE^−/−^ mice (Figure 2F), histological analysis revealed that saringosterol treatment could attenuate the hepatic steatosis in atherosclerotic ApoE^−/−^ mice (Figure 5A). Accordingly, increased liver weight was observed in ApoE^−/−^ mice in the T0 group, while a reduced liver weight was observed in ApoE^−/−^ mice in the SRS group compared to those in the CT group (Figure 5B,C). In agreement with our current knowledge, T0901317 treatment increased the mRNA expression of lipogenic genes, including sterol regulatory element-binding protein-1c (SREBP-1c), acetyl-CoA carboxylase (ACC), fatty acid synthase (FASN), and stearoyl-CoA desaturase 1 (SCD1), a carbohydrate response element binding protein (chREBP) in the liver of ApoE^−/−^ mice (Figure 5D). In contrast, the mRNA levels of these lipogenic genes in the liver of ApoE^−/−^ mice were not increased after saringosterol administration (Figure 5D), further supporting the notion that saringosterol has a selective action for LXRβ. These results together with the above findings suggest that, unlike the pan-LXR agonist T0901317, saringosterol did not cause unfavorable effects on hepatic lipid metabolism in atherosclerotic ApoE^−/−^ mice.

## 3. Discussion

It is known that LXR activation has potent beneficial effects in animal models of atherosclerosis [17]. However, as pan-LXR agonists are able to induce the expression of target genes of both LXRα and LXRβ, they have adverse effects, including hepatic TG accumulation and hypertriglyceridaemia, limiting their therapeutic application [27]. Since it has been accepted that the lipogenic activity of LXRs is primarily mediated by hepatic LXRα, developing or identifying LXRβ-selective agonists has emerged as a strategy to avoid the undesirable lipogenic effects [28]. Previously, we identified saringosterol from edible marine seaweed *Sargassum fusiforme* as a phytosterol with potent and selective action for LXRβ [26]. Here, we evaluated the effects of saringosterol in atherosclerotic ApoE^−/−^ mice. Our results demonstrate that saringosterol treatment could improve lipid profiles and suppress atherosclerosis. Importantly, saringosterol administration did not have those undesirable effects observed after treatment with T0901317, a known pan-LXR agonist. LXRs play important roles in cholesterol homeostasis, as their target genes are critically involved in the regulation of cholesterol metabolism, including absorption, transport, efflux, excretion, and elimination [7]. In agreement with the notion that saringosterol is a potent LXR activator [26], our results suggest that saringosterol treatment might lower the serum cholesterol levels and inhibit atherosclerosis via multiple mechanisms. We found that saringosterol treatment might increase the ABCA1- and ABCG1-mediated cholesterol efflux and inhibit the IDOL-regulated cholesterol uptake in macrophages, enhance the CYP7A1-mediated hepatic cholesterol catabolism and the ABCG5- and ABCG8-mediated excretion of cholesterol from the liver, suppress the NPC1L1-mediated dietary cholesterol absorption from the intestinal lumen, promote the ABCG5- and ABCG8-mediated cholesterol efflux from enterocytes into the intestinal lumen and transintestinal cholesterol excretion, and increase the ABCA1-mediated cholesterol efflux from enterocytes to the circulation. The enhanced reverse cholesterol transport due to the increases in cellular cholesterol efflux, the increased cholesterol elimination and excretion, and the reduced cholesterol absorption might contribute to the cholesterol-lowering and anti-atherosclerotic effects of saringosterol. As saringosterol exhibited beneficial effects similar to those of T0901317, we propose that saringosterol exerts the cholesterol-lowering and anti-atherogenic effects through LXR-mediated pathways.

As LXRα-mediated activation induces hepatic de novo lipogenesis, treatment with pan-LXR agonists will cause hepatic steatosis and hypertriglyceridaemia [18]. In agreement with the notion that saringosterol has a selective action for LXRβ, unlike pan-LXR agonist T0901317 [26], we found that saringosterol treatment did not exacerbate hepatic steatosis in atherosclerotic ApoE^−/−^ mice. Interestingly, attenuated hepatic steatosis and decreased serum TG levels were observed after saringosterol treatment in these atherosclerotic ApoE^−/−^ mice in this study. Although the beneficial effects of LXRβ-selective agonists on lipid homeostasis had been noticed [29], how the beneficial action of saringosterol on serum and hepatic TG levels was achieved in these atherosclerotic ApoE^−/−^ mice in current study require further investigation. Additionally, our mechanistic study revealed that saringosterol might selectively activate the expression of LXR target genes in the liver, intestine, and macrophages. It is worth noting that the expression of LXRα-regulated lipogenic genes was not increased in the liver of ApoE^−/−^ mice after saringosterol treatment. In contrast, the expression of CYP7A1, a direct target gene of LXRs, was increased in the liver of the same mice after saringosterol treatment. However, how the selectivity or specificity of saringosterol for LXRs was achieved in the same tissue of these atherosclerotic ApoE^−/−^ mice also requires further investigation. Nevertheless, our results here demonstrate that saringosterol treatment had favorable rather than adverse effects on the serum and hepatic TG levels in atherosclerotic ApoE^−/−^ mice, suggesting that saringosterol is both safe and effective and could be tested for the treatment of atherosclerosis in the future.

A number of LXRβ-selective agonists have been reported, such as DMHCA [30], tetrachlorophthalimides [31] and 2-oxochromene derivatives [32]. However, neither tetrachlorophthalimides nor 2-oxochromene derivatives has been tested in the atherosclerotic mouse model to evaluate their activity and toxicity. In this study, we evaluated the effects of saringosterol in atherosclerotic ApoE^−/−^ mice and no side effects of hepatic triglyceride accumulation and hypertriglyceridaemia were found. Moreover, several studies indicate that SRS has the beneficial properties of acting as an anti-depressant, acting as an anti-obesity and improving memory in other mouse models.

In conclusion, we showed that SRS has a selective action for LXRβ, improves the homeostasis of cholesterol, and reduces atherosclerosis in a well-established atherosclerosis mouse model, while having no harmful effect on hepatic lipid accumulation and plasma TG levels. Therefore, SRS can be regarded as a promising agent for the prevention of atherosclerosis.

## 4. Materials and Methods

### 4.1. Mice and Treatments

The ApoE^−/−^ mice aged 8 weeks were obtained from Jiangsu Gempharmatech Co., Ltd. (Nanjing, China). The animals were maintained on a 12-h dark/light at the animal facility under specific pathogen-free conditions. After one week of acclimation, mice were randomly divided into three groups (seven mice/group). We fed all mice a high-fat diet (D12108C, Research Diets, Clinton/Cybulsky High Fat Rodent Diet with Regular Casein and 1.25% Added Cholesterol, New Brunswick, NJ 08901, USA) for 12 weeks. Where indicated, dietary interventions by oral administration for the last 2 weeks: the mice in the SRS group were orally treated with 50 mg/kg of saringosterol containing 50% 24(S) epimer (semi-synthesized from commercially available hyodeoxycholic acid) every day. The control group was administered an equivalent volume of phosphate-buffered saline (PBS). As a positive control, mice were treated with T0901317 (71810-50, Cayman, Ann Arbor, Michigan) and the doses were 50 mg/kg/day. At the end of the experiment, animals were sacrificed after fasting for 4 h. Blood was collected, and the serum was separated by centrifugation (4 °C, 3000× *g*, 15 min). Organs (liver, epididymal WAT, ileum) were obtained quickly and stored at −80 °C.

During experiments, mice were anaesthetized with inhaled isoflurane (2%) and were killed using cervical dislocation. All animal protocols were approved by the Animal Care Committee (SIBS-2019-YH-1, date of approval: 28 November 2019, IACUC; SINH-2020-YH-1, date of approval: 1 June 2020, IACUC).

### 4.2. Determination of Serum and Liver Metabolic Parameters

Commercially available kits for the measurement of total-cholesterol (TC) (A113-1-1), low-density lipoprotein-cholesterol (LDL-C) (A113-1-1), high-density lipoprotein-cholesterol (HDL-C) (A112-2-1) and triglyceride (TG) (A110-2-1) were purchased from Nanjing Jiancheng Bio-Engineering Institute Co., Ltd. (Nanjing, China). TC-sensitivity: 6.5 mM; linearity: 0 mM–25.9 mM; inter-assay CV: 3.0%; intra-assay CV: 6.0%. LDL-C-sensitivity: 3.1 mM; linearity: 0.01 mM–10.34 mM; inter-assay CV: 3.0%; intra-assay CV: 8.0%. HDL-C-sensitivity: 1.3 mM; linearity: 0.04 mM–2.59 mM; inter-assay CV: 3.0%; intra-assay CV: 6.0%. TG-sensitivity: 1.7 mM; linearity: 0.01 mM–11.3 mM; inter-assay CV: 3.0%; intra-assay CV: 6.0%.

Serum TC, LDL-C, TG, and the levels of TC, TG in liver were measured by the biochemical kits according to the manufacturer’s instructions. Briefly, 10–20 mg of liver tissue was homogenized in 300 μL isopropyl alcohol and centrifuged supernatants were harvested. The liver extracts and serum were the enzymatically measured with their respective kits.

### 4.3. Aortic Lesion Assessment

The mice hearts were fixed with 4% formaldehyde overnight at 4 °C. Then, specimens were soaked in PBS for 1 h and placed in 30% sucrose overnight. The following day, the hearts were embedded in optimal cutting temperature (O.C.T.) medium and stored at −20 °C. Then, the heart samples were divided into 8-μm sections from the brachiocephalic trunk through the aortic root. Then the aorta root cryosections with the presence of the aorta valve cups were collected. For Oil-red O staining, the aortic root cryostat sections were prepared and stained for lipids with Oil-red O for 20 min at room temperature. After being washed with 60% isopropanol, the sections were counter-stained with hematoxylin. The stained area was quantified using ImageJ (1.8.0) software (National Institutes of Health, Bethesda, MD, USA).

### 4.4. En Face Lesion Analysis

The extent of atherosclerosis development was quantified by en face lesions analysis according to described procedures [33]. After sacrificing mice, we separated the aorta and fixed in 4% paraformaldehyde overnight at 4 °C. After the aorta was washed for 3 min by using 70% ethanol, atherosclerotic plaque was stained for 10 min by using 0.5% Sudan IV (S100286, Aladdin, Shanghai, China) and washed twice for 3 min by using 80% ethanol. The aorta was washed with PBS (pH 7.4). Finally, the adventitial fat was removed and the aorta was opened longitudinally. Digital images were monitored by stereoscopic microscopes and analyzed with the ImageJ (1.8.0) software. The extent of lesions in the entire aorta was calculated, expressed as a percentage of the total aortic surface area covered by lesions.

### 4.5. Immunofluorescence Analysis

CD68 (28058-1, Proteintech, Wuhan, China) expression in arterial lesions was examined by immunofluorescence using 7-mm cryosections of the aortic root. The sections were blocked with 5% bovine serum albumin (BSA) for 1 h and incubated overnight at 4 °C with CD68 antibody (1:100). After washing, the sections were incubated with the Alexa Fluor 594-conjugated goat anti-rabbit IgG secondary antibody (1:200) (A11032, ThermoFisher, Waltham, MA, USA) for 1 h. Nuclei were counterstained with DAPI (1:1000) (C1002, Beyotime, Shanghai, China) for 5 min. The fluorescence signal was monitored by confocal laser scanning microscopy (Zeiss Microsystems, Jena, Germany).

### 4.6. Harvest Mouse Peritoneal Macrophages of ApoE^−/−^ Mice

The mice were placed with abdomen up on paper towel in hood and a small incision was made in the center of the skin overlying the peritoneal wall. The mice were injected with 5 mL of PBS with 5 mM EDTA into the peritoneal cavity carefully. After massaging the abdomen for approximately 10–15 s, approximately 4–4.5 mL fluid was recovered from one mouse. The peritoneal cells were centrifuged (300× *g*, 3 min) and collected, being subsequently cultured in in RPMI at 37 °C with 5% CO_2_ for 6–18 h. During this time, peritoneal macrophages adhered to the plastic surface. The floating non-macrophages were washed away by adding and aspirating 0.5 mL RPMI medium twice. The adherent macrophages were collected.

### 4.7. Cell Culture and Foam Cell Formation

The mouse RAW264.7 macrophages were obtained from the American Type Culture Collection (ATCC, Rockville, MD, USA) and cultured in RPMI 1640 medium (C11875500CP, ThermoFisher, USA) supplemented with 10% fetal bovine serum (FBS) and 2% penicillin/streptomycin (PS) at 37 °C in an atmosphere containing 5% CO_2_. To induce foam cell formation, the cells were incubated with 50 μg/mL ox-LDL (catalog YB-002, Yiyuan Biotechnologies, Guangzhou, China) in serum-free RPMI-1640 medium containing 0.2% bovine serum albumin (BSA) for 24 h. To explore the potential role of SRS, we treated RAW264.7 macrophages-derived foam cells together with SRS (20 μM) or T0901317 (20 μM) for 24 h. All cell experiments were based on the premise of ≥90% cell viability.

### 4.8. Analysis of mRNA Expression

The total RNA was extracted from the livers, intestines, or cells using the RNAiso Plus (9109, Takara, Japan) according to the manufacturer’s protocol. 0.5 µg of RNA was reverse-transcribed into cDNA. cDNA was synthesized with High Capacity cDNA Reverse Transcript Kit (R222-01, Vazyme, Nanjing, China). Real-time PCRs were performed using a commercial SYBR Green Master Mix (11203ES08, YEASEN, Shanghai, China) and QuantStudio 6 real time system. The quantification of gene expression was performed using the comparative cycle threshold (Ct) method. An average Ct value was calculated from the duplicate reactions and normalized to the expression of 18S, and the ΔΔCt value was then calculated. The primers are listed in Table S1.

### 4.9. Statistical Analysis

Data were expressed as means ± SEM. Significant differences were determined by one-way ANOVA analysis followed by Tukey’s multiple comparison test. *p* value of less than 0.05 was considered significant.

## Data Availability

Data are available upon request.

## Supplementary Materials

The following are available online at https://www.mdpi.com/article/10.3390/md19090485/s1, Table S1: List of used primers and their nucleotide sequences.
