# Supplementary material for: Saringosterol from Sargassum fusiforme Modulates Cholesterol Metabolism and Alleviates Atherosclerosis in ApoE-Deficient Mice
Source: Mar Drugs. 2021 Aug 26;19(9):485. doi: 10.3390/md19090485 (PMC8466875; doi:10.3390/md19090485)
Supplement: Supplementary file 1 [file marinedrugs-19-00485-s001.zip › marinedrugs-1349901-supplementary.pdf]

primer	sequence
18S F	*5'-ACCGCAGCTAGGAATAATGGA-3'*
18S R	*5'-CAAATGCTTTCGCTCTGGTC-3'*
ABCA1 F	*5’-GCTTGTTGGCCTCAGTTAAGG-3’*
ABCA1 R	*5’-GTAGCTCAGGCGTACAGAGAT-3*
ABCG1 F	*5’-CTTTCCTACTCTGTACCCGAGG-3’*
ABCG1 R	*5’-CGGGGCATTCCATTGATAAGG-3*
ABCG5 F	*5’-AGGGCCTCACATCAACAGAG-3’*
ABCG5 R	*5’-GCTGACGCTGTAGGACACAT-3’*
ABCG8 F	*5’-CTGTGGAATGGGACTGTACTTC-3’*
ABCG8 R	*5’-GTTGGACTGACCACTGTAGGT-3’*
ACC1 F	*5'-TGTCCACCCAAGCATTTCTTC -3'*
ACC1 R	*5'-CATCCAACACCAGTTCAGTATACGT-3'*
CYP27A1 F	*5'-AGGGCCTCACATCAACAGAG-3'*
CYP27A1 R	*5'-GCTGACGCTGTAGGACACAT-3'*
CYP7A1 F	*5'-GAGAGTGAATCAGGGGACCA-3'*
CYP7A1 R	*5'-TCAGGAATGGAGGGTTTCAG-3'*
CYP7B1 F	*5'-GGAGCCACGACCCTAGATG-3'*
CYP7B1 R	*5'-TGCCAAGATAAGGAAGCCAAC-3'*
CYP8B1 F	*5'-GGGAGTGGGTGGAAGTGAG-3'*
CYP8B1 R	*5'-GTCCTGCATGGATGAAGCT-3'*
chREBP F	*5'-CACTCAGGGAATACACGCCTAC-3'*
chREBP R	*5'-ATCTTGGTCTTAGGGTCTTCAGG-3'*
FASN F	*5'-AAGCGGTCTGGAAAGCTGAA-3'*
FASN R	*5'-AGGCTGGGTTGATACCTCCA-3'*
Hmgcr F	*5’-AGCTTGCCCGAATTGTATGTG-3’*
Hmgcr R	*5’-TCTGTTGTGAACCATGTGACTTC-3’*
IDOL F	*5'-TGTGGAGCCTCATCTCATCTT-3'*
IDOL R	*5'-AGGGACTCTTTAATGTGCAAG-3'*
LDLR F	*5’-TGACTCAGACGAACAAGGCTG-3’*
LDLR R	*5’-ATCTAGGCAATCTCGGTCTCC-3’*
NPC1L1 F	*5'-ATCCTCATCCTGGGCTTTGC-3'*
NPC1L1 R	*5'-GCAAGGTGATCAGGAGGTTGA-3'*
SCD1 F	*5'-CCCTGCGGATCTTCCTTATC-3'*
SCD1 R	*5-TGTGTTTCTGAGAACTTGTGGTG-3'*
SR-B1 F	*5'-CACTACGCGCAGTATGTGCT-3'*
SR-B1 R	*5'-TGAATGGCCTCCTTATCCTG-3'*
SREBP-1c F	*5'-AAGCAAATCACTGAAGGACCTGG-3'*
SREBP-1c R	*5'-AAAGACAAGCTACTCTGGGAG-3'*
SREBP2 F	*5'-GCGTTCTGGAGACCATGGA-3'*
SREBP2 R	*5'-ACAAAGTTGCTCTGAAAACAAATCA-3'*
